# Are Uncultivated Bacteria Really Uncultivable?

**DOI:** 10.1264/jsme2.ME12092

**Published:** 2012-10-10

**Authors:** Indun Dewi Puspita, Yoichi Kamagata, Michiko Tanaka, Kozo Asano, Cindy H. Nakatsu

**Affiliations:** 1 Graduate School of Agriculture, Hokkaido University, N9 W9, Kita-ku, Sapporo, Hokkaido 060–8589, Japan; 2 Bioproduction Research Institute, National Institute of Advanced Industrial Science and Technology (AIST), 2–17 Tsukisamu-Higashi, Toyohira, Sapporo, Hokkaido 062–8517, Japan; 3 Department of Agronomy, Purdue University, West Lafayette, Indiana 47907, USA

**Keywords:** non-dividing cells, yet-to-be-cultivated cells, cultivation efficiency, growth promotion factors

## Abstract

Many strategies have been used to increase the number of bacterial cells that can be grown from environmental samples but cultivation efficiency remains a challenge for microbial ecologists. The difficulty of cultivating a fraction of bacteria in environmental samples can be classified into two non-exclusive categories. Bacterial taxa with no cultivated representatives for which appropriate laboratory conditions necessary for growth are yet to be identified. The other class is cells in a non-dividing state (also known as dormant or viable but not culturable cells) that require the removal or addition of certain factors to re-initiate growth. A number of strategies, from simple to high throughput techniques, are reviewed that have been used to increase the cultivation efficiency of environmental samples. Some of the underlying mechanisms that contribute to the success of these cultivation strategies are described. Overall this review emphasizes the need of researchers to first understand the factors that are hindering cultivation to identify the best strategies to improve cultivation efficiency.

## Introduction

**Fig. 1:**
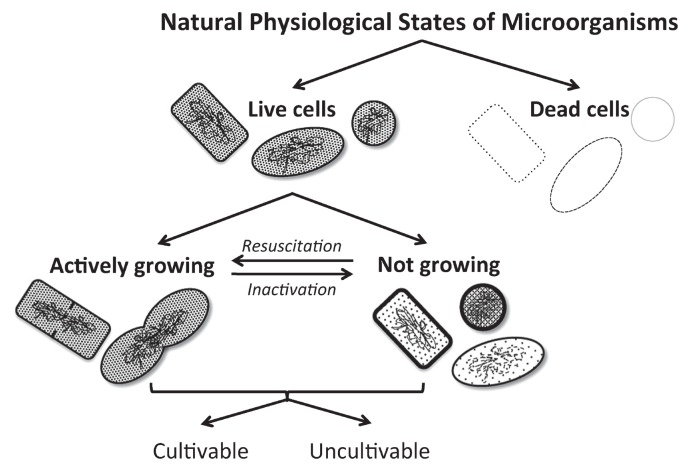
Summarized concepts of typical physiological states of cells. Cells fluctuate between the states of active growth and no growth because of a number of factors that can cause inactivation and resuscitation. Cells from both these states can contribute to the fraction of uncultivable cells in laboratory experiments.

**Fig. 2:**
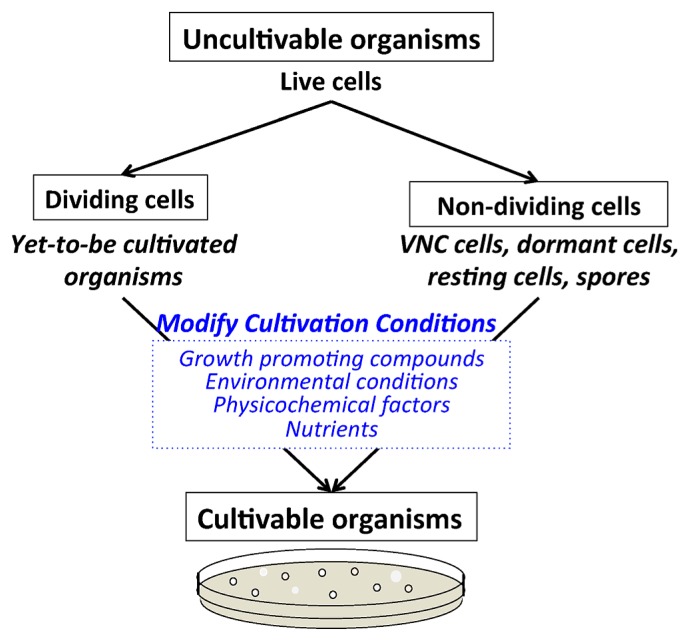
Cultivation strategies used to grow yet-to-be cultivated and non-dividing cells. A fraction of cells collected from natural habitats when transferred to the laboratory can be readily cultivated. In their natural habitat the cells may have been actively growing or in a non-growing state (Fig. 1). The fraction of cells that are uncultivated are categorized in this review as, yet-to-be cultivated and non-dividing cells.

Microorganisms have two basic physiological states, alive and dead (Fig. 1). Living cells in their natural habitat can be actively growing or not growing, typically fluctuating between these two states through inactivation and resuscitation. To gain a better understanding of microorganisms, microbiologists have been cultivating them in the laboratory but have been hampered by the large number of cells that are not readily cultivated (97). The phenomenon of “uncultivable” cells from environmental samples was first observed because of the large discrepancy between cultivable numbers obtained by cultivation (culturing) of cells on agar plates (colony forming units, CFU) versus direct microscopic counts (120). These uncultivable cells fall into two broad categories, (i) bacterial groups with no cultivated representatives (hereafter called yet-to-be-cultivated cells), and (ii) bacteria belonging to groups that have been previously cultivated in the laboratory but whose cells are in a state in which they are alive but no longer replicating (hereafter called non-dividing cells) (1) (Fig. 2). Several bacterial community studies have estimated the percentages of uncultivable cells in various environments (Table 1). In general, the lowest percentages of cultivable cells were obtained from low nutrient environments (*e.g.*, deserts) and the highest from nutrient-rich environments (*e.g.*, fecal samples). In the field of microbial ecology, the importance of yet-to-be cultivated cells to microbial diversity has been well recognized over the past few decades with the development and application of cultivation-independent methods (1, 46). In contrast, the fraction of cells that are uncultivable from environmental samples because they are in a non-dividing state and not readily resuscitated by cultivation has not been extensively studied by microbial ecologists (119, 144). This group of uncultivable cells requires more attention as a means to increase cultivation efficiency from environmental samples. This review covers the basic differences and similarities between these two groups of uncultivable cells and the strategies that have been developed to cultivate them including media, environmental and physiochemical conditions, and the potential contributions of growth promoting compounds from symbiotic interactions. Cultivation of Archaea was not included in this review because of the limited information available on archaeal cell dormancy and strategies for their cultivation. The last section covers some of the underlying mechanisms potentially used or required by cells to initiate growth when various cultivation strategies are used.

## Yet-to-be-cultivated Bacteria

Cells in this category are called uncultivable but in fact they are likely cultivable but currently their appropriate cultivation conditions have not been elucidated (49); therefore these types of uncultivable cells are often called yet-to-be-cultivated cells (126). Although they are not readily cultivated in the laboratory they represent an important group in nature that contributes to microbial diversity and ecosystems (46). Some examples of bacteria included in this category are several subdivisions of *Acidobacteria*, *Verrucomicrobia*, *Proteobacteria* and *Actinobacteria* (105). Strides are being made to cultivate yet-to-be-cultivated cells as indicated by an increase from 13 out of 40 bacterial phyla with cultivated representatives in 1998 (46) to 26 of 52 phyla in 2003 (105). The effort to obtain more cultivated representatives has been made because of the recognition of the important functional role played by these microbes in nature that is still inadequately understood. Cultivation priorities have been made in phyla that are common in some ecosystems but have previously lacked cultivated representatives. For example, the SAR11 clade within alpha Proteobacteria, which is abundant in marine waters (20), and *Verrucomicrobia* and *Acidobacteria* in soil (47) and water (75), and OP10 (124) now have cultivated representatives. By obtaining pure cultures or simple mixed cultures (*e.g.*, syntrophic populations), investigators are able to obtain a deeper understanding of the specific genes related to expressed phenotypes by community members under different biological, chemical and physical conditions (55, 68).

## Cultivation strategies used to grow yet-to-be-cultivated bacteria

Researchers have tried to improve cultivation methods to recover yet-to-be cultivated cells from the environment by providing conditions more reflective of their original habitat. Habitats are influenced by biological, chemical and physical factors and therefore all these factors must be considered when optimizing cultivation conditions (Fig. 2). Some factors are easily measured in the environment and incorporated into the growth strategy, for example, temperature and pH. A limitation has been that many required factors are unknown and difficult to determine without cultivated isolates to test. A number of different strategies have been used to overcome this limitation, (i) development of growth media reflective of the environment of interest, (ii) enrichment of communities in the laboratory using selective conditions of interest, and (iii) enrichment of communities in their natural environment. Additionally the presence of “other organisms” that may have symbiotic relationships with targeted microbes must be considered when developing a cultivation strategy. These “other organisms” may be essential by providing needed growth factors or detrimental by producing growth inhibitors/toxins or competing for resources.

## Modification of growth medium and conditions

Early researchers developed media mainly for the growth of human pathogens (12). The human environment is high in nutrients therefore it was necessary to modify these media for the growth of microbes from other environments, such as water and soil. Strategies such as the use of media with lower nutrient concentrations improves the isolation of marine (20), fresh water (37) and soil bacteria (47) that naturally grow in oligotrophic environments. These media can also be made from natural substrates, for example soil extract medium (69), collected from the habitat of interest to include unknown but essential growth compounds (discussed in the last section of this review) found in that environment. Specific compounds can also be added to media to select microbes with specific functional roles in an environment. This is commonly done to isolate microbes that can utilize specific nutrients (125). A great number of media have been developed for the growth of different microbes and care should be taken to choose the appropriate medium to achieve the objectives of the research being performed.

Even with the use of appropriate growth medium other factors, both physical and biological, must be considered and optimized to improve cultivation efficacy. The objectives for cultivation and the environmental source of material both need to be considered when determining appropriate growth conditions. Although factors such as growth substrate, oxygen availability, temperature and pH are readily manipulated in the laboratory (91), a conscience effort must be made in choosing these conditions. For example, when growing bacterioplankton it may be better to cultivate in liquid culture medium because some of these bacteria do not form colonies at the solid-air interface (29). Although different growth media may be used in an attempt to achieve the high growth efficiency of bacteria from an environmental sample, often multiple growth conditions are not tested (107). This should be considered since most environments undergo fluctuations in nutrient, temperature, pH, and oxygen availability (15, 142). By testing multiple growth conditions researchers have been able to increase cultivation efficiency from freshwater sediment (123).

The biological factors that need to be considered are the physiology of the target bacteria being cultivated and the presence of other organisms that can aid or hinder growth. Many bacteria from oligotrophic conditions have lower growth rates that require longer incubation times to produce visible colonies (49), but less dominant heterotrophs may be more competitively fit under laboratory cultivation conditions and outcompete the oligotrophs. Strategies used to improve the cultivation efficiency of slower growing bacteria are (i) to reduce inoculum size to decrease the chance of encountering competition from smaller populations of faster growing bacteria and (ii) increase incubation time to allow growth to occur (24). The inoculum dilution strategy can also decrease the possibility of exposure to growth inhibitors released by neighboring cells that are producing anti-microbial substances (44). These findings illustrate that to isolate microorganisms from the environment, in addition to the choice of growth media, other factors that should be carefully adjusted are inoculum size and incubation conditions to reflect the physiological needs of the target microbes and environmental conditions in their natural habitat.

## Enrichment of communities in the laboratory

Laboratory enrichment using microcosms is a strategy often used to cultivate cells with specific traits that may not be numerically dominant in a sample. Enrichment enables cells with a specific trait to increase in numbers sufficient for colony formation on agar medium plates. Typically the environmental sample is amended with substrates that will enhance the growth of microbes with the trait of interest. This microcosm can also be incubated in the laboratory under physicochemical conditions reflective of the original natural environment to understand its ecology. For example, a series of different enrichments (63, 92) were performed on soil from a long-term metal-contaminated site (50) that was shown to have a spatially heterogeneous microbial community (11). These microcosms demonstrated the enrichment of different bacterial species depending on the growth substrate, electron donors and acceptors, and the presence of elevated concentrations of lead or chromate (both present at high levels in the soils). This demonstrated the diversity of bacteria present in these highly contaminated soils. Each population constituted a small proportion of the community that flourished when growth conditions were presented that provided them a competitive advantage. A variety of physicochemical conditions likely occur in these soils at different times throughout the year explaining the presence of a variety of populations that likely become dominant depending on the conditions. This demonstrates the value of microcosm enrichment to capture the microbial phylogenetic and functional diversity of certain environments.

## Enrichment of communities in their natural environment

Despite the efforts made in media development and enrichment, the absence of growth factors from specific habitats has still hindered cultivation efforts (13). A more recent strategy being used to ensure the presence of these growth factors is to incubate cells in their natural environment using a diffusion chamber (13, 51), hollow fiber membrane chamber (5), or soil slurry membrane system (33). These incubation systems allow the distribution of growth factors from the original habitat into the media through a membrane while preventing the accessibility of cells from outside the system. This method differs from using extracts made from the habitat because microbes on the other side of the barrier can continually produce unstable or readily degraded growth factors. This strategy has increased the cultivation efficiency as well as the diversity of the microorganisms obtained. For example, using a diffusion chamber, ten different phyla of bacteria were cultivated including two phyla with few cultivated representatives (13). Instead of using filters to separate cells, another strategy being used is the encapsulation of single cells in gel microdroplets that are then exposed to the environment, allowing the exchange of molecules such as nutrient, metabolites and growth factors through the gel with no direct contact with other cells (139). Flow cytometry can then be used to sort gel microdroplets containing colonies after growth occurs. A broad range of marine bacteria, including previously uncultivated bacteria, has been isolated using this technique with a microtiter plate continuously fed filtered seawater. These results show that the presence of compounds from natural habitats in growth media is required to increase the cultivation efficiency of many yet-to-be-cultivated microbial taxa.

## Non-dividing cells: Groups with cultivated representatives

The second group of uncultivated cells has been called viable but not culturable (VNC) or non-dividing cells. These cells were first recognized because they were being cultivated in the laboratory but due to unknown factors they were no longer dividing yet remained alive (138). VNC is defined as a state in which cells are alive but no longer growing by forming visible colonies on solid media or increasing turbidity in liquid media routinely used for their cultivation (98). Also in this category there are cells in environmental samples that remain dormant or do not begin growing in the laboratory despite having representatives that have been previously cultivated under laboratory conditions (119). It has been suggested that the term VNC is an oxymoron because growth has commonly been used as a measure of viability (7) therefore alternative terms have been used, such as, non-dividing, resting, and dormant cells (56, 58). In the following sections we describe factors that have contributed to cells entering a non-dividing state, the morphological and genetic characteristics of cells in this state, and factors that have been found that have aided in the resuscitation of these cells into a dividing state.

## Non-dividing cells: a resting stage

Most ecosystems in nature and in the laboratory can be unstable, exposing bacteria to a variety of stresses that can be unfavorable for their growth (107). To survive stressful conditions one strategy used by bacteria is to enter a dormant or resting state and resume growth once conditions become more favorable (28). Although spores are the most studied form of resting cells they are not the focus of this manuscript because many reviews are already available on all aspects of sporulation (25, 32, 43). Instead we will focus on non-spore forming bacteria that can enter a similar resting state. The majority of research in this group has been on “viable but not culturable” food and waterborne pathogens (98, 99) because of health concerns: however, bacteria in this state also contribute to the uncultivable fraction of cells from most natural habitats in laboratory cultivation attempts. It is important to know their characteristic features to recognize cells in this state since they likely play important roles in ecosystem functions when they are in a viable state (29, 31, 66).

The formation of non-dividing cells by non-spore forming bacteria has been documented in a number of Gram-negative and Gram-positive bacteria (99). There are many commonalities between the resting stage of spore forming and non-spore forming bacteria. Stress factors such as alterations in nutrients, temperature and oxygen (112) as well as pH (114), and high osmotic pressure (100) have been shown to trigger non-spore forming bacteria to enter a non-dividing state. Studies have been conducted to understand the underlying mechanisms involved in the entry of cells into the non-dividing state (45). In this state cells have been shown to survive extremely adverse conditions and for prolonged periods (128). Similar to spore-forming cells, the non-dividing state might be one of the strategies used by non-spore forming bacteria for survival under unfavorable conditions.

## Morphological characteristics of non-dividing cells

Although endospores are not formed, non-spore forming bacteria also exhibit altered morphological features (Table 2) when they are in a non-dividing state that contributes to their survival. An important morphological characteristic of non-dividing cells is the change in cell wall structure and composition compared to actively growing cells. Thickening of cell walls has been observed in non-dividing Gram-positive bacteria *Mycobacterium* species (22) and in Gram-negative *Vibrio cholerae* cells (61). Furthermore, chemical analysis of the cell wall has shown an increase in 3→3 peptidoglycan crosslinks in non-dividing cells of both Gram-negative and Gram-positive bacteria, including *Escherichia coli* (117), *Mycobacterium tuberculosis* (65) and *Enterococcus faecalis (*118). The increase of 3→3 cross-links is thought to increase resistance of these cells to hydrolytic activity (65) and to make some species more resistant to mechanical disruption (118). A lower concentration of mycolic acid has been observed in non-dividing *Mycobacterium smegmatis* cells that potentially causes a decrease in cell wall permeability (109), which may contribute to their survival by decreasing uptake of antibiotics and chemotherapeutic agents (72). These findings show that despite the lack of a spore wall, modifications to the wall of non-dividing cells contribute to survival during dormancy.

Another important morphological characteristic of non-dividing cells is changes in cell membrane composition. In Gram-negative bacteria, increased saturated fatty acid composition in the phospholipid layer has been observed in *Pseudomonas aureofaciens* (59), *V. cholerae* (40), *V. vulnificus* (71), and *E. coli* (71). An increase in saturated fatty acid composition in the phospholipid layer can lead to a decrease in membrane fluidity (89) as observed in non-dividing *Micrococcus luteus* cells (86). Adjustment of membrane fluidity under stress is an important mechanism to maintain cytoplasmic membrane integrity, which is vital for cell viability (89). Although the benefit of decreased membrane fluidity for the survival of non-dividing cells remains to be elucidated, these observations indicate that there is a relationship between this morphological change and the dormant state.

## Genetic characteristics of non-dividing cells

One of the genetic characteristics of endospores is that the integrity of chromosomal DNA is preserved and becomes functional when exiting the resting stage (104). Similarly, DNA preservation was also observed in non-dividing cells of non-spore forming bacteria. When observed by electron microscopy, nucleoids of non-dividing cells appeared to be more compact than actively growing cells (19, 86), but others have reported that the DNA content was not significantly changed in some cells (86) or decreased over time in other cells (133). Endogenous metabolism and protection of cells from stress conditions is essential for dormant cells to retain their viability (116). The DNA of non-dividing cells has been shown to remain functional; the expression of several genes has been reported in cells in the non-dividing state (6, 60). For example, in non-dividing *M. tuberculosis* cells, genes involved in cell wall biosynthesis were up regulated while those involved in energy production and conversion were down regulated (60). In non-dividing *V. cholerae* cells, up to 100 genes were induced, including genes responsible for DNA metabolism and other genes involved in essential cellular processes (6). Of most importance is the maintenance of DNA repair mechanisms to ensure genomic DNA is not totally degraded during the stationary phase (70); however, we should note that mutations occur more frequently in stationary cells but this is an important means of increasing genetic diversity that can lead to beneficial mutations and can provide cells with a competitive advantage when resuscitated (34).

## Resuscitation of cells from a “non-dividing” state

To confirm the viability of non-dividing cells it is important to demonstrate that they have the potential to resume growth (99). This process proceeds through two stages, reactivation from the resting stage by a resuscitation factor followed by cell division. Bacterial communication compounds such as pheromones or cytokines can act as signals to initiate growth but in some cases resuscitation can be achieved simply by the removal of stress factors. For example, in the case of cell starvation, a simple remedy could be the restoration of nutrients (28). Another factor to consider is the need to remove compounds contributing to oxidative stress, for example H_2_O_2_, which can cause cellular damage. The loss of catalase activity in *V. vulnificus* when incubated at low temperature resulted in VNC cells that were sensitive to H_2_O_2 (_62). This resulted in growth inhibition when these cells were transferred to nutrient-rich agar medium and optimum temperature because of oxidative stress damage from accumulation of superoxide and free radicals. The addition of pyruvate or catalase to culture medium has been shown to restore cell growth (81, 96). In contrast to simple modifications of media it is more challenging to resuscitate cells if specific growth factors are needed (28). In the first section of this review we discussed a number of cultivation strategies that are also applicable to the growth of non-dividing cells. In the following section we review growth factors produced by bacteria that have been successfully used to cultivate non-dividing cells. These factors may also be involved in the growth of yet-to-be-cultivated bacteria, especially when using growth conditions that mimic the natural environment.

## Growth promotion factors and their use in cultivation

Signaling compounds involved in growth stimulation have been called growth factors, autocrine growth factors, cytokines and growth-promoting factors (58). Addition of these growth factors to culture medium has been shown to resuscitate non-dividing cells in the laboratory (Table 3) and increase cell numbers grown from environmental samples (Table 4). The growth factors that have been studied most extensively are peptidoglycan fragments, cyclic adenosine monophosphate (cAMP), and N-acyl homoserine lactone (AHLs). Many studies testing growth factors have been performed using pathogenic bacteria because of the significant role they play in the resuscitation, growth and virulence of clinical bacteria (28, 57), but growth factors used for the resuscitation of non-dividing cells are not limited to pathogens. By understanding the potential growth factors required for resuscitating bacteria from a non-dividing state they can be incorporated into cultivation strategies to increase cultivation efficiency from environmental samples.

### Peptidoglycan fragments and a resuscitation-promoting factor (Rpf)

Fragmented cell wall peptidoglycans have been shown to act as a signaling molecule to initiate growth in both-spore forming (110) and non-spore-forming bacteria (53). Enzymatically fragmented cell wall peptidoglycans are released during exponential growth (28) for use in new cell wall synthesis (27), but these fragments have also been shown to be a signal for *Bacillus subtilis* endospore germination. *B. subtilis* spores sense the signaling molecule from neighboring cells when the peptidoglycan fragments bind to the extracellular PASTA domain of serine/threonine protein kinase, PrkC, resulting in activation of the intracellular kinase domain to form phosphorylated elongation factor G (EF-G) that is believed to play an important role in spore germination (110).

A similar peptidoglycan fragment-mediated mechanism has been found in *Mycobacterium* exiting dormancy (53). Several Actinobacteria including *M. tuberculosis* produce a resuscitation-promoting factor (Rpf), a muralytic enzyme that hydrolyzes peptidoglycan (56). Rpf cleaves β-1-4-glycosidic links between *N*-acetylmuramic acid (MurNAc) and *N*acetylglucosamine (GlcNAc) to produce peptidoglycan fragments (84). In *M. tuberculosis*, peptidoglycan fragments have been shown to bind to the extracellular PASTA domain of Ser/Thr kinase PknB, a homolog of PknC in *B. subtilis*, which resulted in an increase in the cultivability of non-dividing cells (79). Rpf as a resuscitation factor has also been documented in a number of non-pathogenic Gram-positive bacteria, such as *Micrococcus luteus* (85), *M. smegmatis (*112), and *Rhodococcus rhodochrous* (113). These results suggest that peptidoglycan turnover and cell wall remodeling in Gram-positive bacteria are important factors in cell resuscitation from dormancy.

In studies of natural habitats, peptidoglycans were found to comprise a large fraction of dissolved organic matter in rivers (48), sea water (90), and marine sediments (103). In the laboratory, in Gram-negative bacteria such as *E. coli*, 60% of the peptidoglycan is recycled every generation and the recovery rate is efficient, resulting in very small amounts of peptidoglycan remaining in the culture medium (101). In contrast, large amounts of peptidoglycan fragments are detected in culture medium of spore-forming Gram-positive bacteria during the exponential growth phase (27), leading to the question, “Why is there differential utilization of peptidoglycans by bacterial species?” Although the major sources of peptidoglycans and turnover mechanism in the environment remain unclear there is evidence that there is differential degradation of Gram-positive and Gram-negative cell walls. Comparison of two killed marine isolates, *Pseudomonas* sp. and *Bacillus* sp., revealed greater degradation of the Gram-positive walls in estuarine waters (48), suggesting that the peptidoglycan fragments derived from Gram-positive bacteria have functions in the environment that require further exploration.

### Cyclic adenosine monophosphate (cAMP)

Cyclic adenosine monophosphate (cAMP) is a molecule in signaling pathways important for regulating a variety of cellular processes in response to environmental changes in prokaryotic and eukaryotic organisms (76). The involvement of cAMP in catabolism was first demonstrated in *E. coli* but it is now known to be involved in regulating many functions (14, 102). In bacteria, cAMP is the second messenger in signaling pathways; it is synthesized from ATP by adenylate cyclase (AC) after its activation by an environmental signal (14, 76). The cAMP then activates an effector, most commonly the cAMP receptor protein (CRP) (102). Details of various effectors and regulatory mechanisms are reviewed in greater detail elsewhere (14, 76). Most relevant to this review is its involvement in the regulation of a number genes (36) including *rpf* genes (106).

In nature, dissolved cAMP has been reported in freshwater (35) and seawater (2). The uptake of cAMP from seawater by marine bacterioplankton corresponded to bacterial abundance and activity, suggesting that cAMP was related to their metabolism and growth (2). Furthermore, uptake of radiolabeled cAMP by means of specific high-affinity transport systems into marine bacteria supported the hypothesis that cAMP uptake contributed to the intracellular pool of this regulatory compound (3). The addition of cAMP to growth medium has been reported to increase the cultivation efficiency of marine and freshwater bacterioplankton (15–17). From a seawater sample, 100% cultivation efficiency was reported for bacterioplankton determined by the most probable number (MPN) method in comparison to the DAPI total cell count (15). In addition to increasing cultivation efficiency, using the MPN method, addition of cAMP to growth medium was found to result in the cultivation of bacterioplankton from a eutrophic lake that were previously uncultivated (17). Furthermore, a test of the growth-promotion activity of cAMP on one marine isolate revealed an increase in growth rates and biomass, suggesting that it may serve as a signaling compound for resuscitation of non-dividing cells in the marine ecosystem (15). Based on the variety of regulatory systems activated by cAMP it is a prime candidate for consideration as an additive to media to increase cultivation efficiency.

### *N*-acyl homoserine lactone (AHLs)

Bacteria use signaling compounds to communicate with other cells using quorum sensing (8, 9, 78, 132). A number of different quorum-sensing signaling molecules are involved in a variety of cellular functions. The first and most extensively studied quorum-sensing signal is *N*-acyl homoserine lactone (AHL) found in Gram-negative bacteria (8, 9, 78, 132). Others have extensively reviewed this topic and for the purposes of this review only a few characteristics will be emphasized from these papers (8, 9, 78, 132). AHL is produced intracellularly by LuxI and released from the cell. The extracellular concentration of AHL increases with increasing population density and can diffuse back into cells. When the signaling compound accumulates above a threshold level it binds to an intracellular receptor, LuxR or a homologue. The number and variety of genes regulated by quorum sensing support the need to incorporate this knowledge when developing cultivation strategies.

Addition of AHL compounds to culture media has been shown to increase viable counts of bacteria isolated from environmental samples. For example, addition of *N*-(butyryl)-dl-homoserine lactone (BHL) and *N*-(oxohexanoyl)-dl-homoserine lactone (OHHL) to growth media increased the cultivation efficiency of marine bacteria from the Baltic Sea (15). Addition of AHL mixtures to growth media increased the MPN value of heterotrophic bacteria from lake water (18) and the frequency of Acidobacteria detected from soil (121). AHLs have also been reported to stimulate growth and colonization in biofilm (77), decrease the lag phase of growing bacteria after starvation (10), influence siderophore synthesis (39), control catalase and superoxide dismutase production (42), and regulate starvation survival (38). The mechanism of non-dividing cell resuscitation by AHLs in natural environments remains to be elucidated but is believed to be related to the regulation of genes associated with the growth function of quorum sensing.

### Other growth factors

Many other growth factors have been reported to resuscitate non-dividing cells in the laboratory although the mechanistic basis remains unclear; the following are a few examples. A low concentration of oleic acid (1 μg mL^−1^) can resuscitate non-dividing *M. smegmatis* cells (93). YeaZ protein (94) from Gram-negative bacteria was used to resuscitate non-dividing *Salmonella enterica* serovar Oranienburg (100). An 8 kDa peptide (Rv1174c) from *M. tuberculosis* could resuscitate non-dividing cells (140). In environmental samples, 5-amino-acid peptides of LQPEV were identified as a growth promotion compound used to cultivate previously uncultivable cells from marine sediment (95). These findings illustrate the variety of compounds that can stimulate growth after exiting dormancy and require more in-depth study, with the likelihood that many more growth factors exist.

## Conclusion

The phenomenon of uncultivable bacterial cells from environmental samples has resulted from our inadequate understanding of the conditions necessary for their growth and not because they are truly uncultivable. The uncultivable group is comprised of yet-to-be-cultivated cells (from groups with no cultivated representatives) and non-dividing cells (from groups with cultivated representatives). Improvement in cultivation efficiency has been achieved by incorporating knowledge of the chemical, physical and biological variables of natural habitats into cultivation strategies. Great strides have been made by modifying nutrient content of growth medium, adjustment of incubation condition, removal of growth-limiting factors and using growth environments more reflective of the original habitat. Also, there is now a better understanding of some of the underlying mechanisms that has made these cultivation strategies more efficient, such as different signaling compounds produced by cells to promote or initiate growth. In the future, by building on our current knowledge, the phenomenon of uncultivable cells may be eliminated. Attention should be paid particularly to using cultivation conditions more reflective of the natural habitat and gaining a greater understanding of growth promotion factors produced by cells for incorporation into cultivation strategies.

## Figures and Tables

**Table 1:** The cultivation efficiency of cells from various environmental samples

Habitat	Cultivation efficiency^a^ (%)	Media for cultivation^b^	Method for direct counts^c^	Reference
Desert	0.0007–0.02	R2A	PLFA	(21)
	0.02–0.15	R2A	PLFA	(67)
	0.05–0.1	TSA	PLFA	(67)
Permafrost sediment	0.001–10	Poor and rich media	AO	(128)
	0.03	R2A	SYTO9/PI	(41)
Heavy metal-contaminated soil	0.08–2.27	R2A	DTAF	(30)
	0.03–1.48	TSBA	DTAF	(30)
Soil	2.4–19	VL55	DAPI, AO	(108)
Marine sediment	2.5^M^	ABW	DAPI	(15)
Sea water	0.25^M^	ABW	DAPI	(15)
	0.003	Marine R2A	DAPI	(122)
	0.01–0.98	1/10 Marine R2A	DAPI	(20)
	0.01–0.15	Marine R2A	DAPI	(20)
Lake sediment	0.1	PE03-7A	EtBr	(123)
	1.3	PE03-7G	EtBr	(123)
	0.007–0.017	ABM	SYTO9/PI	(111)
Fresh water	0.1–5.59^M^	Synthetic fresh water	DAPI	(17)
Activated sludge	13.86	LB agar	DAPI	(130)
	0.24–0.38	TSA	DAPI	(52)
	1.67–3.68	R2A	DAPI	(52)
Human feces	54	Medium 10	DAPI	(74)
	14.28	BBA	DAPI	(64)
	36.5	BBA	DAPI	(64)
	58	Medium 10	n.r.^d^	(135)

a Cultivation efficiency was calculated from the percentage of cultivable cells from colony forming units or MPN counts^M^ in proportion to total number of live cells from direct counts.

b Cultivation was performed aerobically except for human feces that were grown anaerobically.

c Direct counts were made microscopically after staining with AO (Acridine orange), DAPI (4′,6-diamidino-2-phenylindole), DTAF (5-(4,6-dichlorotriazin-2-yl)aminofluorescein), ethidium bromide (EtBr) or SYTO9/PI (SYTO9 and propidium iodide) except for estimations made from phospholipid fatty acid (PLFA) analysis.

d not reported

**Table 2:** Characteristics of non-dividing cells^a^ of non-spore forming bacteria

Parameters	Characteristics	References
Cell size	Size reduction	(109)
Cell morphology	Change in cell shape (*e.g.* cyst-like, coccoid) and organization (*e.g.* cell wall thickening, altered outer electron-dense layer, increase periplasmic space, membrane curling)	(4, 22, 61, 88)
Cell membrane phospholipid	Reduction of phosphatidylglycerol, increase of cardiolipin content, and increase in saturated fatty acid	(40, 60, 71, 86)
Electrochemical properties	Increase of particle conductivity (due to thickening of cell wall) and decrease of electrochemical activities	(143)
Ribosome	Reduction of ribosomes	(86)
DNA	Poorly visible nucleoid but DNA still present	(86, 88, 133)
Metabolic activity	Reduction of metabolic activity, membrane oxydase activity, and dehydrogenase activity	(54, 86, 113)
Total cell protein	Reduction of cytoplasmic protein	(86, 109)
Fatty acid	Reduction of fatty acid content and change in lipid composition	(71, 93)

a Characteristics differences are in respect to actively growing cells of non-spore forming bacteria.

**Table 3:** Compounds used to resuscitate non-dividing cells of several non-spore forming bacteria induced in the laboratory.

Compounds	Microorganism	Method used to induce cells into non dividing state	References
H_2_O_2_ degrading compound	*Vibrio cincinnatiensis*	Starvation, low temperature	(141)
	*Vibrio parahaemolyticus*	Starvation, low temperature	(80, 136)
	*Vibrio vulnificus*	Starvation, low temperature	(134)
	*Vibrio cholerae*	Starvation, low temperature	(6)
	*Enterococcal* species	Starvation, low temperature	(73)
	*Escherichia coli* O157:H	Starvation, low temperature	(81)
	*Aeromonas hydrophila*	Starvation, low temperature	(131)
Rpf^a^	*Micrococcus luteus*	Starvation	(54, 82, 83, 85, 129)
	*Mycobacterium tuberculosis*	Starvation, hypoxic	(113, 115, 137)
	*Mycobacterium smegmatis*	Starvation	(112)
	*Rhodococcus rhodochrous*	Starvation	(113, 127)
	*Arthrobacter globiformis*	Starvation	(26, 87)
Lipid	*Mycobacterium smegmatis*	Starvation	(93)
Peptide	*Mycobacterium tuberculosis*	Starvation	(140)
YeaZ protein	*Salmonella enterica* serovar Oranienburg	Osmotic stress	(100)

a resuscitation promoting factor

**Table 4:** Compounds tested to increase cell numbers grown from environmental samples collected from various habitats

Compounds	Habitat	Specific taxa reported^a^	Reference
cAMP^b^	Sea water and sediment	Strain G100, New Rhodobacteraceae	(15)
	Eutrophic lake	Two new Actinomycetales	(17)
AHL^c^	Sea water and sediment	No specific taxa reported	(15)
Peptidoglycans	Estuarine water	No specific taxa reported	(48)
Peptide	Intertidal sand sediment	*Psychrobacter* sp. MSC33	(95)
Siderophore	Intertidal sand sediment	*Maribacter polysiphoniae* KLE1104	(23)
		*Cyclobacterium sp.* KLE1009	
		*Sulfitobacter sp.* KLE1123	
		*Maribacter sp.* KLE1063,	
		*Winogradskyella sp.* KLE1078	
		*Hyphomonas sp.* KLE1080	
		*Reinekea sp.* KLE1125	
		*Simiduia sp.* KLE1111	
		*Sulfitobacter sp.* KLE1123	

a Specific taxa noted by authors that apparently depended on the presence of the added compound for growth in laboratory culture medium. In most cases taxa names were for most closely related identified species but more in-depth identification may demonstrate that these taxa represent new genera.

b cyclic adenosine monophosphate

c N-acyl homoserine lactone
